# Prediction of pancreatic atrophy after steroid therapy using equilibrium‐phase contrast computed tomography imaging in autoimmune pancreatitis

**DOI:** 10.1002/jgh3.12316

**Published:** 2020-03-10

**Authors:** Yasutaka Yamada, Atsuhiro Masuda, Keitaro Sofue, Eisuke Ueshima, Hideyuki Shiomi, Arata Sakai, Takashi Kobayashi, Takuya Ikegawa, Shunta Tanaka, Ryota Nakano, Takeshi Tanaka, Maya Kakihara, Shigeto Ashina, Masahiro Tsujimae, Kohei Yamakawa, Shohei Abe, Masanori Gonda, Shigeto Masuda, Noriko Inomata, Hiromu Kutsumi, Tomoo Itoh, Takamichi Murakami, Yuzo Kodama

**Affiliations:** ^1^ Division of Gastroenterology, Department of Internal Medicine Kobe University Graduate School of Medicine Kobe Japan; ^2^ Department of Internal Radiology Kobe University Graduate School of Medicine Kobe Japan; ^3^ Centor for Clinical Research and Advanced Medicine Establishment Shiga University of Medical Science Ostu Japan; ^4^ Diagnostic Pathology Kobe University Graduate School of Medicine Kobe Japan

**Keywords:** autoimmune pancreatitis, diabetes mellitus, equilibrium‐phase images, pancreatic atrophy

## Abstract

**Background and Aims:**

Imaging tools for predicting pancreatic atrophy after steroid therapy in autoimmune pancreatitis (AIP) have not been established. As delayed equilibrium‐phase contrast enhancement in computed tomography (CE‐CT) may reflect interstitial fibrosis, we evaluated the ability of equilibrium‐phase CT imaging for predicting pancreatic atrophy.

**Methods:**

Forty‐six steroid‐treated AIP patients who underwent contrast‐enhanced CT at our university hospital were included in this retrospective study. CT attenuation (Hounsfield units [HU]) values in noncontrast images (NC) and equilibrium‐phase images (EP) and the differences in HU values between NC and EP images (SUB) were measured. Pancreatic volume was measured in CE‐CT before (Vol_pre_) and after (Vol_post_) steroid therapy. The volume reduction rate was calculated. The relationships of CT values with pancreatic atrophy, Vol_post_, volume reduction rate, and diabetes exacerbation were investigated.

**Results:**

CT values in the EP and SUB images before steroid therapy were associated with pancreatic atrophy after steroid therapy (atrophy *vs* nonatrophy 114.5 ± 12.8 *vs* 99.5 ± 11.1, *P* = 0.0002; 70.9 ± 14.72 *vs* 57.2 ± 13.1, *P* = 0.003, respectively), but CT values in NC images were not (*P* = 0.42). CT values in EP and SUB images before steroid therapy were correlated with Vol_post_ (EP images *r* = −0.70, *P* = 0.002; SUB images *r* = −0.68, *P* = 0.03) and volume reduction rate after steroid therapy (EP images: *r* = −0.55, *P* < 0.0001; SUB images *r* = −0.45, *P* = 0.002). Diabetes exacerbation was associated with higher EP and SUB values (*P* = 0.009 and *P* = 0.04, respectively).

**Conclusion:**

Equilibrium‐phase contrast CT imaging may facilitate prediction of pancreatic atrophy after steroid therapy in AIP.

## Graphical abstract

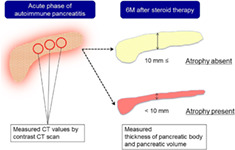

Pancreatic atrophy is often observed after steroid therapy for AIP and is associated with exacerbation of diabetes. However, it has not been possible to predict pancreatic atrophy before the start of therapy. This time, we have found a method to predict pancreatic atrophy using CT values, and we introduce it here.

## Introduction

The pathogenesis of autoimmune pancreatitis (AIP) is assumed to involve autoimmune mechanisms with unknown etiology.^1^ Given its immune‐related mechanisms, steroid therapy is recommended as a first‐line therapy in the International Consensus Diagnostic Criteria for AIP and the Japanese Clinical Guidelines for AIP.^2^ In cases showing steroid resistance or unacceptable conditions, immune‐modulatory agents such as antimetabolites and anti‐CD20 antibody are considered alternatives.^3^, ^4^, ^5^

Nevertheless, the influence of steroid therapy on diabetes control in AIP remains controversial. Some reports have indicated that steroid therapy worsens diabetes control.^6^, ^7^ In contrast, other reports have indicated that 25–45% of AIP patients exhibited improved diabetes control with steroid therapy.^8^, ^9^ Previously, we reported that pancreatic atrophy was observed in one‐third of AIP patients 6 months after the beginning of steroid therapy. Pancreatic atrophy after steroid therapy was closely related to the incidence of new onset of diabetes or exacerbation of diabetes, and most patients required insulin therapy even in the maintenance phase of AIP.^10^ However, the predictors of pancreatic atrophy before steroid therapy remain unknown. Prediction of pancreatic atrophy before steroid therapy will enable identification of patients who should receive early therapeutic intervention for diabetes or nonsteroid therapy, such as immune‐modulatory agents.

Delayed contrast enhancement in the equilibrium phase of contrast‐enhanced computed tomography (CE‐CT) has been reported to reflect interstitial fibrosis in the liver and pancreas.^11^, ^12^, ^13^, ^14^ Histopathologically, AIP is characterized by a dense infiltrate of IgG4‐positive plasma cells around the small and large interlobular ducts, accompanied by fibrosis and atrophy of the acini.^15^ However, the predictive ability of equilibrium‐phase contrast CT imaging for pancreatic atrophy in AIP has not yet been examined. In this study, we hypothesized that equilibrium‐phase contrast CT imaging in AIP patients would reflect pancreatic fibrosis before steroid therapy and predict pancreatic atrophy after steroid therapy. In order to test this hypothesis, we utilized a database of 46 consecutive AIP patients who were treated with steroid therapy and evaluated the ability of equilibrium‐phase CT imaging in predicting pancreatic atrophy after steroid therapy.

## Methods

### *Patients*

From December 2005 to December 2017, 63 consecutive patients were diagnosed with AIP at our hospital. The diagnosis of AIP was based on the Japanese Clinical Guidelines for AIP.^7^ All patients were categorized as having type 1 AIP. Of the 63 patients, 46 were treated with steroid therapy and were enrolled in this study. According to the Japanese Clinical Guidelines for AIP, prednisolone at an initial dosage of 30 mg/day was administered for 2–4 weeks. The dose was then tapered by 5 mg every 2–4 weeks until the dosage for maintenance therapy was attained. Maintenance therapy at a dosage of 2.5–7.5 mg/day was continued thereafter. Medical records were examined, and information on demographics (age and gender), alcohol intake (>50 g/day), serum IgG4 level, current smoking, and diabetes was collected. The patterns of pancreatic swelling (diffuse or focal/segmental) and other organ involvement were also examined by imaging studies, including CT scans. This study was conducted in accordance with the Declaration of Helsinki and its amendments. The study protocol was reviewed and approved by the ethics committee at our university. All authors had access to the study data and reviewed and approved the final manuscript.

### *CT protocol*

CT examinations were performed using multidetector‐row CT systems (Brilliance 16; Philips Medical Systems, Best, Netherlands. Aquilion 64 or One; Canon Medical Systems, Otawara, Japan). Although detailed scan parameters (e.g. detector configuration, gantry rotation, table pitch) were modified according to age, essential parameters (tube voltage, slice thickness/reconstruction interval, acquisition timing of the equilibrium phase images) were identical throughout the study period.

Precontrast images were first obtained, and triple‐phase dynamic contrast‐enhanced CT was acquired with a tube voltage of 120 kVp. Iodinated contrast material was administered into an antecubital vein using an automatic power injector at a dose of 510mgI/kg, and the arterial phase (45‐s delay), portal venous phase (70‐s delay), and equilibrium phase (150‐s delay) were obtained for all patients.

### *Evaluation of pancreatic atrophy*

**Figure 1:**
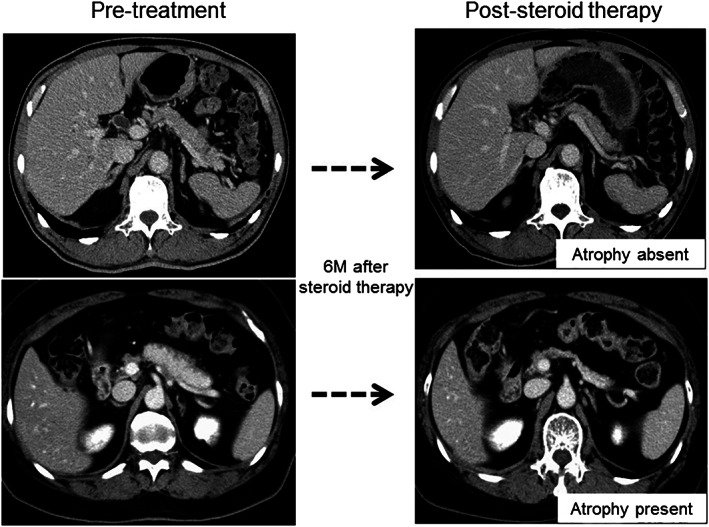
Typical computed tomography images of atrophy or nonatrophy cases before and after steroid therapy

Pancreatic atrophy was evaluated 6 months after the beginning of steroid therapy (the end of first steroid therapy term). Pancreatic atrophy after steroid therapy was defined as being present when the thickness of the pancreatic body was less than 10 mm, as previously described.^10^ Typical images of atrophy or nonatrophy cases are shown in Figure 1. Then, pancreatic volume was measured in CE‐CT before (Vol_pre_) and after (Vol_post_) steroid therapy. Two board‐certified expert radiologists (K.S. and E.U.) measured pancreatic volume by consensus in all patients by using commercially available software (Ziostation® 2 Type1000; Ziosoft Inc., Tokyo, Japan). Pancreatic volume was calculated by multiplying the operator‐defined area of the pancreas in each image slice by the interval thickness and by summing each volume in individual slices, avoiding dilated main pancreatic ducts, focal lesions, and coarse calcification if present. The pancreatic reduction rate was then calculated by dividing Vol_post_ by Vol_pre_.

### *Evaluation of CT values with equilibrium‐phase contrast CT imaging*

**Figure 2:**
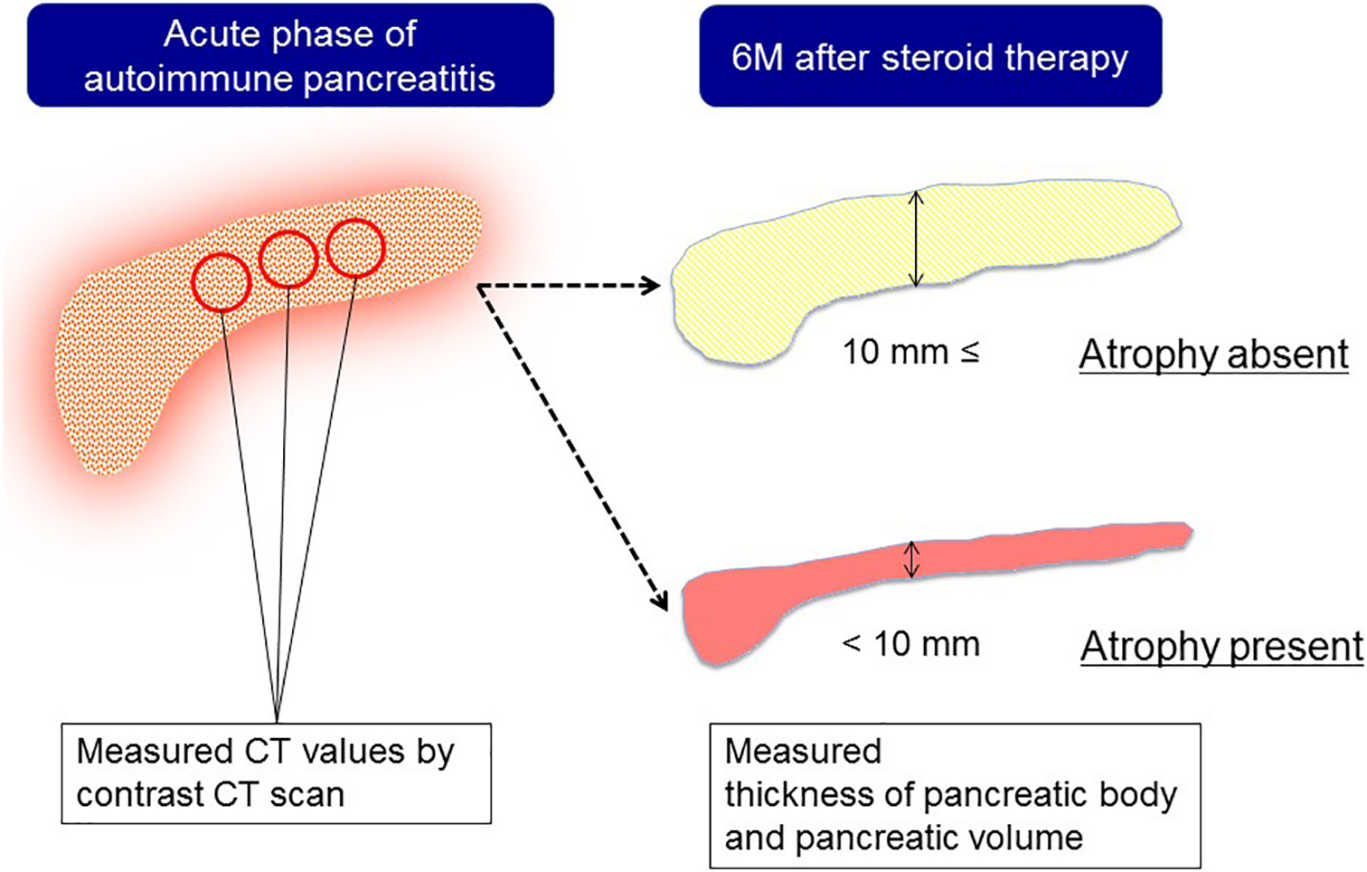
The computed tomography (CT) value was calculated from the average value of three arbitrary regions of interest set in the enlarged pancreatic parenchyma. The relationships of CT values with pancreatic atrophy were evaluated. The presence or absence of atrophy was judged by a body diameter of 10 mm

Triple‐phase dynamic CT images were obtained before steroid therapy in all patients. Precontrast images were initially acquired, and arterial phase, portal venous phase, and equilibrium‐phase images were obtained after intravenous administration of iodinated contrast material. Two board‐certified expert radiologists (K.S. and E.U.) measured CT attenuation (Hounsfield units [HU]) values in noncontrast (NC) and equilibrium‐phase (EP) images by consensus by identifying three regions of interest (ROIs).The ROIs were placed, as large as possible (minimum: 100 mm^2^), on the swelling lesion of the pancreas, avoiding dilated main pancreatic duct and large vasculature with regard to the arterial and portal venous phase images as well. To maintain the reproducibility of the measured CT values, the ROIs were placed three times on each lesion, and average values were recorded for all patients. The calculated HU values were averaged for each image. Associations of pancreatic atrophy after steroid therapy with the following three parameters were investigated: (i) HU values in NC images, (ii) EP images, and (iii) difference between HU values in NC and EP images (SUB images). The relationship of each CT value with pancreatic volume (Vol_post_) and volume reduction rate after steroid therapy was also investigated (Fig. 2).

### *Evaluation of diabetic status*

Diabetes mellitus was diagnosed according to the diagnostic criteria of the Japan Diabetes Society.^16^ The HbA1c values were expressed in accordance with the National Glycohemoglobin Standardization Program (NGSP) guidelines.^17^ A decrease in HbA1c by more than 0.5% in patients treated with diet therapy and the same dose or decreased dose of oral antidiabetic agents and/or insulin therapy was considered to indicate an improvement after steroid therapy. An increased dose or initial use of insulin/antidiabetic agents, increased HbA1c level by more than 0.5%, or new onset of diabetes was considered exacerbation of diabetes. Other patterns were considered to indicate absence of changes.

### *Statistical analysis*

Statistical analyses were conducted using Stata/SE 12.1 for Macintosh (StataCorp, College Station, TX). The distribution of data was evaluated with the *χ* ^2^ test or Fisher's exact test when the number of expected cells was less than five. Normally distributed variables were compared using Student's *t*‐test. Nonnormally distributed variables were compared using the Wilcoxon rank sum test. For correlation analysis of pancreatic volume (Vol_post_) and pancreatic reduction rate with each CT value (NC, EP, and SUB values), as well as correlation of pancreatic volume (Vol_post_) and pancreatic body thickness, Spearman's rank correlation test was used. *P* values less than 0.05 were considered to be statistically significant.

## Results

### *Patient characteristics according to pancreatic atrophy*

Steroid therapy was effective in all treated patients (*n* = 46). Six months after commencing steroid therapy, pancreatic atrophy was observed in 14 patients but not in the remaining 32 patients. Mean thickness of the pancreas body before steroid therapy was 16.39 ± 4.64 mm in patients with atrophy and 18.57 ± 3.69 mm in those without atrophy. Mean thickness of the pancreas body 6 months after the beginning of steroid therapy was 7.51 ± 1.89 mm in patients with pancreatic atrophy and 14.7 ± 3.85 mm in those without atrophy. There was no association of pancreatic atrophy after steroid therapy with age, gender, other organ involvement, pattern of pancreas swelling, serum IgG4 levels, current smoking, or alcohol intake (Table 1).

**Table 1:** Patient characteristics according to pancreatic atrophy

	Total	Atrophy† present (*N* = 13)	Atrophy† absent (*N* = 33)	*P* value
Age (yr, mean ± SD)	46	68.3 ± 9.73	62.7 ± 14.8	0.20
Gender				0.62
Male	35	10	25	
Female	11	4	7	
Other organ involvement				0.31
Bile duct	5	2	3	
Retroperitoneal fibrosis	6	2	4	
Salivary/Iacrimal glands	12	4	8	
Others	12	2	10	
None	7	3	4	
Pattern of pancreas swelling				0.61
Diffuse	32	9	23	
Segmental/focal	14	5	9	
Pretreatment serum IgG4 (mg/dl, mean ± SD)	46	346.2 ± 218.6	557.3 ± 485.1	0.14
Current smoking				0.33
Presence	15	6	9	
Absence	31	8	23	
Alcohol intake (more than 50 g/day)				0.97
Presence	10	3	7	
Absence	36	11	25	

† Pancreatic atrophy 6 months after steroid therapy was defined to be present when the thickness of the pancreas body was less than 10 mm.

### *Relationship between pancreatic volume and pancreas body thickness*

Pancreas body thickness (less than 10 mm) is a simple and useful marker of pancreatic atrophy, as previously described.^10^ However, it is unknown whether pancreas body thickness reflects the entire pancreatic volume. Therefore, we investigated the relationship of pancreas body thickness with pancreatic volume and volume reduction rate. Pancreas body thickness before and after steroid therapy was correlated with pancreatic volume before and after steroid therapy (before steroid therapy *r* = 0.31, *P* = 0.047; after steroid therapy *r* = 0.60, *P* < 0.0001; Supplemental Fig. S1a,b). Pancreatic volume reduction rate was also correlated with pancreatic body thickness after steroid therapy (*r* = −0.49, *P* = 0.001; Supplemental Fig. S1c). For the diagnosis of pancreatic atrophy, the cutoff value of pancreatic volume after steroid therapy (Vol_post_) and the volume reduction rate were 22.7 mm^3^ and 51.6%, respectively.

### *Relationship between pancreatic atrophy and CT values in equilibrium‐phase contrast CT imaging*

**Figure 3:**
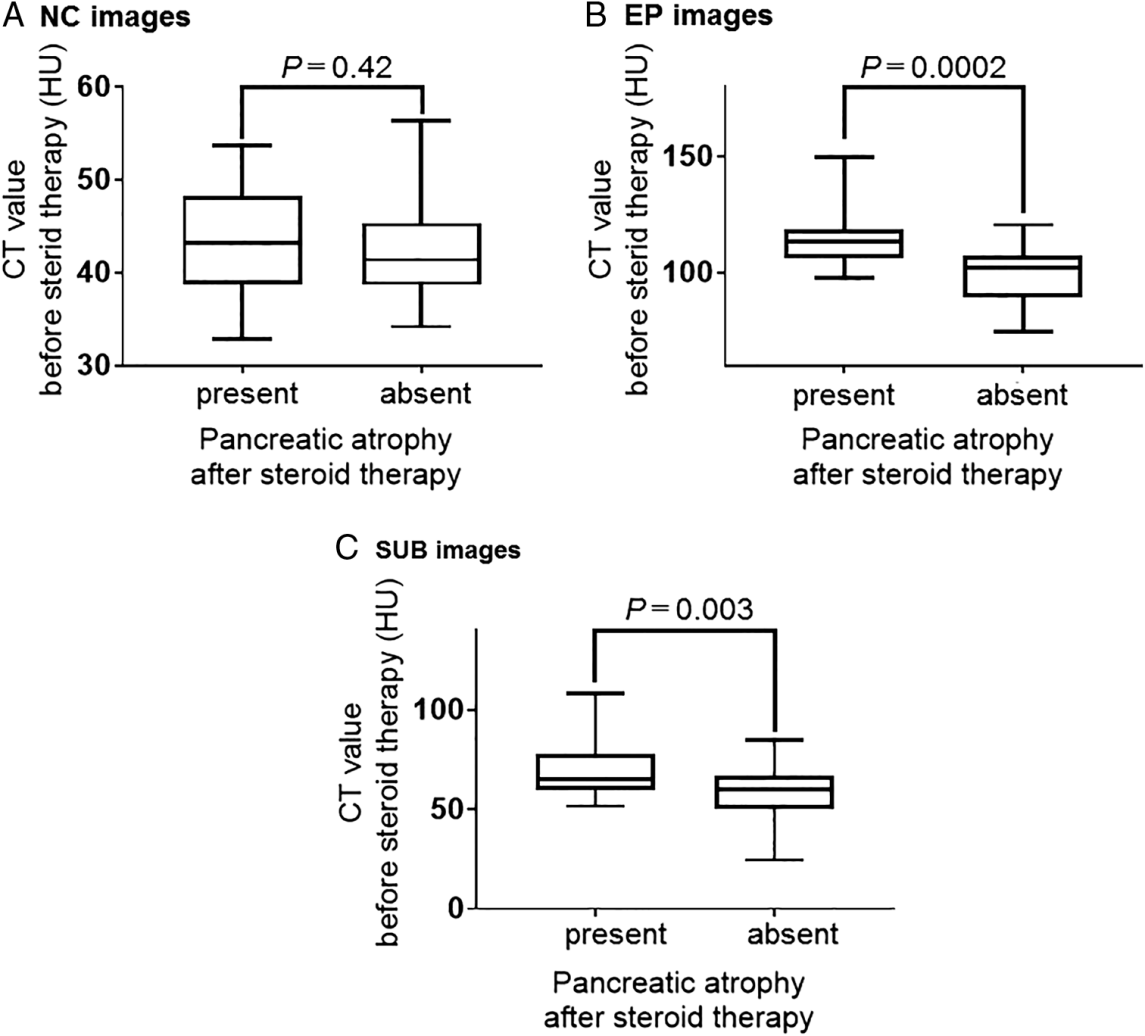
Associations between pancreatic atrophy after steroid therapy and computed tomography values in the NC, EP, and SUB images

The associations between pancreatic atrophy after steroid therapy and CT values in the NC, EP, and SUB images were investigated (Fig. 3). There were no significant differences in HU values of NC images between AIP patients with atrophy and those without atrophy (43.6 ± 6.0 *vs* 42.2 ± 5.0, *P* = 0.42, Fig. 3a). CT values of EP and SUB images in AIP patients with atrophy were higher than those in AIP patients without atrophy (114.5 ± 12.8 *vs* 99.5 ± 11.1, *P* = 0.0002, and 70.9 ± 14.7 *vs* 57.2 ± 13.1, *P* = 0.003, respectively; Fig. 3b,c).

### *Relationship between pancreatic volume after steroid therapy and the CT values in equilibrium‐phase contrast CT imaging*

**Figure 4:**
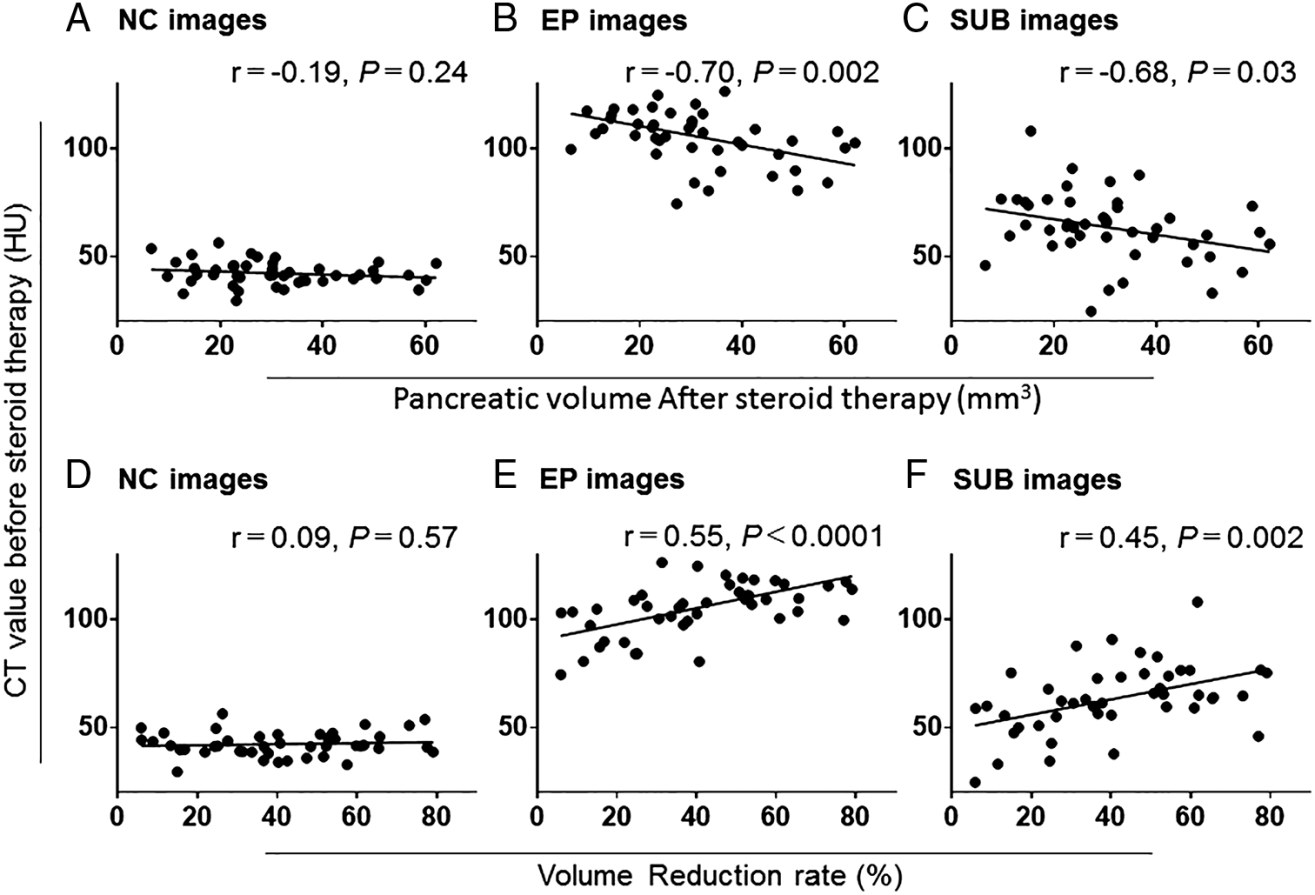
Associations of volume reduction rate with computed tomography values in NC, EP, and SUB images before steroid therapy

The associations of pancreatic volume after steroid therapy (Vol_post_) with CT values in NC, EP, and SUB images before steroid therapy were investigated (Fig. 4a–c). There were no significant associations of HU values of NC images with Vol_post_ of whole pancreas (NC image *r* = −0.19, *P* = 0.24; Fig. 4a). Vol_post_ was associated with HU values in EP and SUB values (*r* = −0.70, *P* = 0.002 and r = −0.68, *P* = 0.03, respectively; Fig. 4b,c).

### *Relationship between volume reduction rate and CT values of equilibrium‐phase contrast CT imaging*

The pancreatic volume reduction rate due to steroid therapy was calculated by dividing the pancreatic volume after steroid therapy (Vol_post_) by that before steroid therapy (Vol_pre_). The associations of volume reduction rate with CT values in NC, EP, and SUB images before steroid therapy were investigated (Fig. 4d–f). There were no significant associations between the HU values of NC images and the volume reduction rate of the whole pancreas (NC image *r* = 0.09, *P* = 0.57; Fig. 4d). Volume reduction rate was associated with EP and SUB values (*r* = 0.55, *P* < 0.0001 and *r* = 0.45, *P* = 0.002, respectively; Fig. 4e,f).

### *Relationship of exacerbation of diabetes with pancreatic atrophy, pancreatic volume, volume reduction rate, and CT values*

Among 46 steroid‐treated AIP patients, 13 showed exacerbation of diabetes. The associations of exacerbation of diabetes with pancreatic atrophy, pancreatic volume, volume reduction rate, and CT values were evaluated (Table 2). Cutoff values for each parameter were calculated by receiver operating characteristic analysis based on the association with pancreatic atrophy. The cutoff values for pancreatic volume and pancreatic volume reduction rate were 22.7 mm^3^ and 51.6%, respectively. The cutoff values for EP and SUB were 109.2 HU and 63.9 HU, respectively. Exacerbation of diabetes was associated with a thin pancreas body, lower pancreatic volume after steroid therapy, and higher pancreatic volume reduction rate (presence or absence of exacerbation of diabetes: 69.2% [9/13] *vs* 15.2% [5/33], *P* = 0.0003; 61.5% [8/13] *vs* 15.2% [5/33], *P* = 0.002; and 76.9% [10/13] *vs* 21.2% [7/33], *P* = 0.0006, respectively). The exacerbation of diabetes was associated with higher EP and SUB values (presence or absence of exacerbation of diabetes: 69.2% [9/13] *vs* 27.3% [9/33], *P* = 0.009 and 69.2% [9/13] *vs* 36.4% [12/33], *P* = 0.04, respectively).

**Table 2:** Relationships between the exacerbation of diabetes and CT parameters

	Exacerbation of diabetes† present (*N* = 13)	Exacerbation of diabetes† absent (*N* = 33)	*P* value
Pancreatic atrophy§			
Thickness of pancreatic body <10 mm	69.2% (9/13)	15.2% (5/33)	0.0003
Pancreatic volume < 22.7 mm^3^ ‡	61.5% (8/13)	15.2% (5/33)	0.002
Pancreatic volume reduction rate 51.6 ≦ (%)‡	76.9% (10/13)	21.2% (7/33)	0.0006
CT values before steroid therapy			
EP images 109.2 ≦ (HU)‡	69.2% (9/13)	27.3% (9/33)	0.009
SUB images 63.9 ≦ (HU)‡	69.2% (9/13)	36.4% (12/33)	0.04

† The exacerbation of diabetes included an increased dose or initial dose of insulin/antidiabetic agents, an increase in HbA1c level by more than 0.5%, or new onset of diabetes.

‡ Cutoff value of each parameter was calculated by ROC analysis based on the association of pancreatic atrophy.

§ Pancreatic atrophy after steroid therapy was defined to be present when the thickness of the pancreas was less than 10 mm.

CT, computed tomography; EP, equilibrium phase; ROC, receiver operating characteristic; SUB, subtracted HU values.

## Discussion

The present study demonstrated that CT values in equilibrium‐phase contrast CT imaging may be useful for predicting pancreatic atrophy after steroid therapy. Furthermore, CT values in equilibrium‐phase contrast CT imaging were related to the reduction rate of pancreatic volume and could also predict the exacerbation of diabetes after steroid therapy. To the best of our knowledge, this is the first study to assess the predictive ability of imaging tools for pancreatic atrophy after steroid therapy in AIP. Predicting pancreatic atrophy before steroid therapy could help identify high‐risk cases for the exacerbation of diabetes.

Our previous report demonstrated that pancreatic atrophy after steroid therapy was related to the exacerbation and new onset of diabetes mellitus in AIP.^10^ However, in both previous and current studies, it was difficult to predict pancreatic atrophy using patients' pretreatment factors, including pretreatment serum IgG4 levels, swelling pattern of the pancreas at diagnosis, and pancreas body thickness before treatment (Table 1). In atrophy cases, many patients exhibited pancreatic atrophy soon after the beginning of steroid therapy (within 6 months after steroid therapy). Therefore, we hypothesized that histopathological differences existed, including acinar atrophy or intestinal fibrosis, between atrophy and nonatrophy cases during the diagnosis of AIP (even in the acute phase of AIP), although patients' pretreatment factors could not distinguish them.

In previous reports, magnetic resonance imaging (MRI) has been mainly used to investigate the relationship between pancreatic fibrosis and imaging studies.^18^, ^19^ Multiparametric MRI of the pancreas, including imaging with the T2‐corrected Dixon technique and intravoxel incoherent motion diffusion‐weighted imaging, may yield quantitative information regarding pancreatic steatosis and fibrosis.^19^ The pancreas‐to‐muscle signal intensity ratio on in‐ and opposed‐phase T1‐weighted images could be a potential biomarker for pancreatic fibrosis and elevated HbA1c values.^20^ However, there is scant evidence of an association between pancreatic fibrosis and CT images. There is only one report evaluating pancreatic fibrosis by contrast‐enhanced CT.^11^ In AIP, evidence to evaluate the association between pancreatic fibrosis and CT images is lacking. In comparison with MRI, contrast‐enhanced CT is essential for the diagnosis of AIP and is most commonly used in several institutions. Furthermore, there are small differences in measurement values in each CT model relative to those in MRI.^21^ In this study, CT values of both EP and SUB images were correlated with pancreatic atrophy after steroid treatment, suggesting that CT values could be a widely acceptable fibrosis marker for AIP patients.

Steroid therapy for AIP has a remarkably high remission rate of 96–100% and may be accepted as first‐line standard treatment.^22^, ^23^, ^24^ AIP mainly occurs in the elderly, and steroids have various side effects, including lumbar compression fracture, femoral head necrosis, and impaired glucose tolerance.^2^, ^22^ Therefore, in some patients, such as those with premetabolic syndrome including prediabetes, long‐term steroid therapy may be harmful. In some countries, antimetabolites and anti‐CD20 antibody are also being considered treatment options.^3^, ^4^, ^5^ Equilibrium‐phase contrast CT imaging could predict atrophy after steroid therapy and identify cases at high risk of exacerbation of diabetes. In patients at high risk of exacerbation of diabetes, a nonsteroid therapeutic agent may be warranted.

This study had several limitations. First, there was no evidence of an association between pathological pancreatic atrophy or fibrosis and CT findings. Recently, the usefulness of endoscopic ultrasound‐guided fine needle aspiration (EUS‐FNA) for the diagnosis of AIP has been reported.^25^, ^26^, ^27^, ^28^ Indeed, in the majority of cases showing focal swelling, we performed EUS‐FNA upon diagnosis of AIP. In the present cases, we attempted to elucidate the association between pathological fibrosis and CT images by using EUS‐FNA specimens. However, the samples were very small, were inadequate for evaluating the state of the whole pancreas, and did not correlate with pancreatic atrophy (data not shown). Second, this was a retrospective study, and the sample size was limited. Further large‐scale prospective studies are necessary to verify our results.

In conclusion, equilibrium‐phase contrast CT imaging findings during the diagnosis of AIP could be a predictor for pancreatic atrophy after steroid therapy. Equilibrium‐phase contrast CT imaging could reflect pancreatic fibrosis and predict pancreatic atrophy, which were related to the exacerbation of diabetes in AIP patients.

## Supporting information


**Figure S1** The relationship of pancreas body thickness with pancreatic volume and volume reduction rate.
