# Supplementary figures and images for: Prediction of pancreatic atrophy after steroid therapy using equilibrium‐phase contrast computed tomography imaging in autoimmune pancreatitis
Source: JGH Open. 2020 Mar 10;4(4):677–83. doi: 10.1002/jgh3.12316 (PMC7411657; doi:10.1002/jgh3.12316)

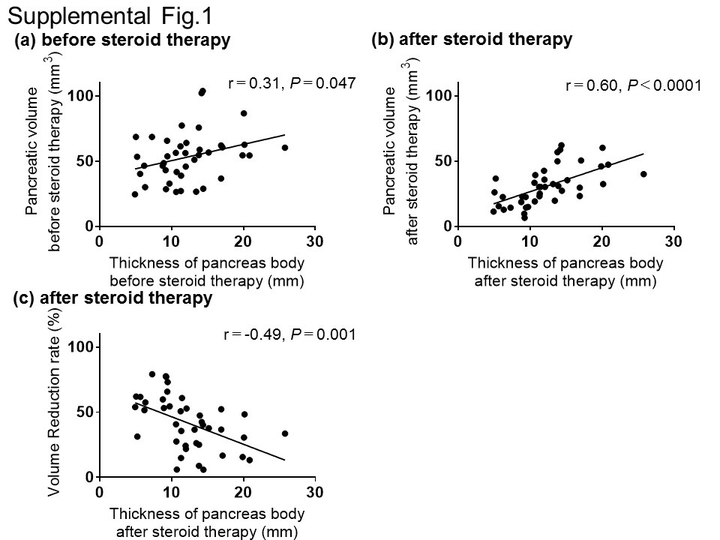

Supplement: Supplementary file 1 — Figure S1 The relationship of pancreas body thickness with pancreatic volume and volume reduction rate. [file JGH3-4-677-s001.jpg]
